# Which Clinical Factors Are Associated with the Post-Denosumab Size Reduction of Giant Cell Tumors? The Korean Society of Spinal Tumor (KSST) Multicenter Study 2023-02

**DOI:** 10.3390/cancers17132121

**Published:** 2025-06-24

**Authors:** Min Wook Joo, Se-Jun Park, Wanlim Kim, Yongsung Kim, Jae Hwan Cho, Nicholas Matthew Bernthal, Minpyo Lee, Jewoo Lee, Yong-Suk Lee

**Affiliations:** 1 Department of Orthopaedic Surgery, St. Vincent’s Hospital, College of Medicine, The Catholic University of Korea, Seoul 06591, Republic of Korea; 2 Department of Orthopaedic Surgery, University of California Los Angeles, Los Angeles, CA 90095, USA; 3 Department of Orthopedic Surgery, Spine Center, Samsung Medical Center, School of Medicine, Sungkyunkwan University, Seoul 06351, Republic of Korea; 4 Department of Orthopedic Surgery, Asan Medical Center, College of Medicine, University of Ulsan, Seoul 05505, Republic of Korea; 5 Department of Orthopaedic Surgery, Seoul National University Bundang Hospital, College of Medicine, Seoul National University, Seoul 13620, Republic of Korea; 6 Department of Orthopaedic Surgery, Incheon St. Mary’s Hospital, College of Medicine, The Catholic University of Korea, Seoul 06591, Republic of Korea

**Keywords:** giant cell tumor of bone, denosumab, neoadjuvant treatment

## Abstract

Background/Objectives: Denosumab has been considered effective for downstaging giant cell tumor of bone (GCTB), implying that it lowers the Campanacci grade rather than reducing tumor size. Preoperative tumor shrinkage holds therapeutic value by decreasing surgical complications. While previous studies have observed lesion shrinkage in some patients, no study has identified the types of patients likely to show treatment response. Thus, we sought to identify the clinical factors associated with post-denosumab GCTB size reduction. Methods: The data for 45 GCTB patients (29 females [64%], 16 males [36%], median age 32 years [range: 17–65]) who received denosumab were analyzed. Lesions were in the extremities (*n* = 25, 56%) or spinopelvic region (*n* = 20, 44%). Ten (22%) were recurrent. Lesion size reduction was assessed using two criteria: a ≥5% and a ≥5 mm decrease in the longest diameter. Univariate analyses were conducted for all variables, and those found to be significant were subjected to multivariate analyses. In addition, multicollinearity was evaluated. Subgroup analyses were performed based on lesion location and recurrence status. Results: Campanacci grade III predicted proportional shrinkage (≥5%) in all patients (OR 4.819, 95% CI 1.121–20.714) and in extremity (OR 11.171, 95% CI 1.023–122.014) and primary lesions (OR 5.781, 95% CI 1.181–28.297), and aneurysmal bone cyst (ABC)-like change was associated with absolute shrinkage (≥5 mm) in all patients (OR 8.734, 95% CI 1.159–65.845) and primary lesions (OR 11.936, 95% CI 1.074–132.69). The longest tumor diameter of ≥7 cm predicted absolute shrinkage in all patients (OR 12.380, 95% CI 1.038–147.694) and for spinopelvic lesions (OR 20, 95% CI 1.676–238.63). Conclusions: ABC-like change, Campanacci grade III, and the longest tumor diameter might predict post-denosumab GCTB shrinkage in all patients, though shrinkage varies with lesion location and recurrence status. These factors could help clinicians tailor treatment strategies in different settings. Further research is needed to explore how clinical factors pharmacologically influence denosumab-induced GCTB shrinkage.

## Simple Summary

Denosumab is known to reduce the size of giant cell tumor of bone (GCTB) under some conditions, which is a therapeutic effect distinct from downstaging. However, the clinical predictive factors associated with such a response remain uncertain. This study aimed to identify predictors of post-denosumab tumor shrinkage in various clinical GCTB settings. Campanacci grade III was a significant predictor of shrinkage in the overall cohort, and in patients with extremity or primary lesions. Aneurysmal bone cyst-like change was associated with size reduction in the overall cohort and in patients with primary tumors. Additionally, a large baseline tumor size predicted shrinkage in all patients and those with spinopelvic GCTBs. Recognizing these factors may help clinicians better anticipate response to denosumab and guide treatment strategies tailored to individual patients.

## 1. Introduction

Denosumab is a monoclonal antibody that targets receptor activator of nuclear factor-kappa B ligand (RANKL) used to treat giant cell tumors of bone (GCTB) [1,2,3]. It has been considered an effective agent for the surgical downstaging of GCTB in a neoadjuvant setting [3]. However, surgical downstaging does not refer to reducing tumor size but rather to lowering the Campanacci grade, more specifically, from grade III to grade II, which reflects the reduction and disappearance of cortical destruction and soft tissue involvement [4]. Meanwhile, size reduction per se may also have therapeutic value, as it can reduce surgical complexity and lessen the need for extensive reconstruction. It may also help prevent iatrogenic neurovascular complications in general, and, in particular, intraoperative spinal cord and neural plexus injuries in GCTBs located in axial regions. These complications can occur not only during surgical dissection but also due to intraoperative adjuvant treatments using liquid nitrogen, phenol, hydrogen peroxide, anhydrous ethanol, electrocautery, polymethylmethacrylate, and argon plasma cautery [5].

Previous studies [3,6,7,8] have reported a size reduction in GCTB in some patient groups using image criteria such as those referred to in Response Evaluation Criteria in Solid Tumors version (RECIST) [9] and by the European Organization for Research and Treatment of Cancer (EORTC) [10]. In addition, tumor response has been assessed using inverse Choi density/size (ICDS) [11], which evaluates size reduction as well as changes in density and signal intensity.

Nevertheless, no study has been conducted to determine which patient groups experience lesion shrinkage. Thus, we asked: Which clinical factors are associated with post-denosumab GCTB size reduction in different settings?

## 2. Materials and Methods

### 2.1. Study Design and Setting

This multi-center retrospective observational study was approved by the Institutional Review Board of St. Vincent’s Hospital, The Catholic University of Korea (VC23RIDI0250). Data on patients treated with denosumab for GCTB at St. Vincent’s Hospital, Samsung Medical Center, Asan Medical Center, and Seoul National University Bundang Hospital from January 2018 to November 2023 were collected, and relevant medical records, including demographic and clinical characteristics, along with imaging studies were investigated.

### 2.2. Participants

**Figure 1:**
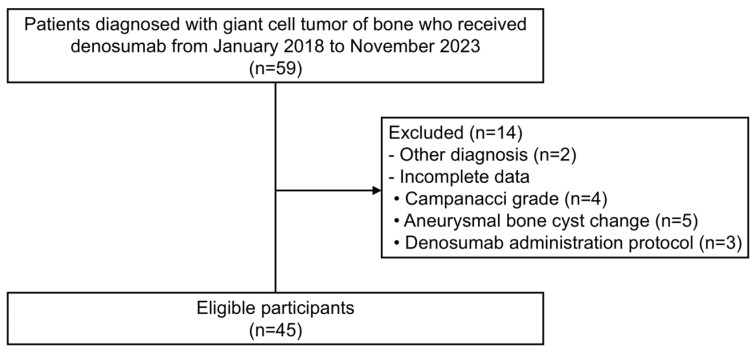
This Strengthening the Reporting of Observational Studies in Epidemiology (STROBE) flowchart describes cohort selection.

Initially, we considered 59 patients with GCTB, who had received denosumab between January 2018 and November 2023 at one of four tertiary centers. However, two patients were excluded due to other diagnoses, and 12 patients were excluded due to incomplete data: missing Campanacci grade (*n* = 4), aneurysmal bone cyst (ABC)-like change (*n* = 5), or details of denosumab administration protocol (*n* = 3). Finally, 45 patients were included in the study (Figure 1). Given the exploratory nature of this study, a sample size estimation was not considered.

### 2.3. Variables and Outcome Measures

We considered several variables, including sex, age, lesion location, Campanacci grade, ABC-like change, lesion recurrence, extraosseous extension, denosumab protocol compliance, number of denosumab injections, and the pre-denosumab longest tumor diameter. Age groups were defined using a cutoff of 40 years (<40 and ≥40). Lesions were categorized based on the extremity and spinopelvic region. Campanacci grade was assessed based on plain radiographs. Lesions exhibiting both soft-tissue involvement and a fuzzy tumor border were classified as grade III, whereas those lacking both features were categorized as grade I or II. Grade I and II lesions were defined by an intact cortex and by cortical thinning or mild expansion, respectively [4]. Extraosseous extension and ABC-like change were confirmed by magnetic resonance imaging (MRI). Lesion recurrence was defined as primary or recurrent. As regards denosumab protocol compliance, the standard protocol was defined as 120 mg every 4 weeks, with additional doses of 120 mg on days 8 and 15 during the first month [2]. Any deviation from the regimen was regarded as non-standard. Numbers of denosumab injections were classified as ≥5 or <5. The longest tumor diameters were measured in axial MRI view by a single orthopedic surgeon at each participating institution, all of whom are coauthors of this study, and classified as ≥7 cm, 5–7 cm, or <5 cm.

For the analysis, proportional and absolute decrease in tumor size were defined as decreases of ≥5%, or ≥5 mm, respectively, in the longest diameter. As lesions with a longer pre-denosumab tumor diameters may show substantial absolute reductions despite small proportional changes, both criteria were considered. The cutoffs for lesion shrinkage were selected by exploring multiple thresholds and evaluating whether a significant predictive factor appears.

### 2.4. Statistical Analysis

For the descriptive analysis, continuous variables are presented using medians and ranges (minimum–maximum) because they did not follow a normal distribution, while categorical variables are described with numbers and percentages. Logistic regression analysis was performed to estimate odds ratios (ORs) and 95% confidence intervals (CIs), and to identify variables associated with the longest tumor diameter size reduction. All variates were subjected to univariate analysis to select candidate predictors of tumor size reduction (*p* < 0.1) [12], and candidate variables were subsequently subjected to multivariate analysis to identify independent predictors (*p* < 0.05). Multicollinearity among covariates was assessed using the variance inflation factor (VIF). Variables with a VIF > 10 were considered to have high collinearity. Less significant variables among them were excluded from the multivariate analysis.

In addition, subgroup analyses were performed on extremity and spinopelvic lesions and on primary and recurrent lesions.

## 3. Results

### 3.1. Predictive Factors of Tumor Size Reduction in All Patients

The demographic and clinical characteristics of the study participants (female: 29 [64.4%]; male: 16 [35.6%]) are presented in Table 1. Median participant age was 32 years (range: 17–65 years). Twenty-five (55.6%) and 20 (44.4%) lesions were located in either the extremity or spinopelvic region, respectively. Nine were in the upper extremity: four in the distal radius, three in the proximal radius, one in the proximal humerus, and one in the distal ulna. Sixteen were in the lower extremity: seven in the distal femur, three in the proximal tibia, three in the proximal fibula, one in the proximal femur, one in the cuneiform, and one in the calcaneus. Regarding the 20 spinopelvic GCTBs, most were vertebral except for one (5%) ischial lesion. Thirty patients followed a standard denosumab protocol, while fifteen patients underwent a non-standard protocol. Among the fifteen patients with the non-standard protocol, seven had altered dosing intervals and eight skipped one or more induction doses. Median longest tumor diameter for the 45 lesions was 4.55 cm (range: 1.92–11.8 cm), and denosumab was injected a median of five times (range: 3–19 times). The median interval between the last denosumab administration and MRI checkup was 6 months (range: 1–88 months). Table 1 also presents potential predictive factors associated with GCTB size reduction, and related outcomes; for all 45 patients, 19 (42.2%) achieved proportional lesion shrinkage and 15 (33.3%) had absolute shrinkage.

The results of predictive factor analyses are shown in Table 2. A female sex, Campanacci grade III, and ≥5 denosumab injections were identified as candidate predictors of proportional shrinkage in univariate analyses, but only Campanacci grade III was significant by multivariate analysis (OR, 4.819; CI, 1.121–20.714).

Lesion shrinkage of ≥5 mm was associated with ABC-like change, number of denosumab injections (≥5), and the longest tumor diameter of ≥7 cm by univariate analysis. Multivariate analysis showed the significant predictive factors were ABC-like change (OR, 8.734; CI, 1.159–65.845) and the longest tumor diameter being ≥7 cm (OR, 12.380; CI, 1.038–147.694).

### 3.2. Subgroup Analyses on Predictive Factors by Lesion Location

The results of additional analyses conducted to identify factors associated with reduction in the sizes of extremity and spinopelvic lesions are presented in Table 3 and Table 4, respectively. Univariate analyses for extremity lesions showed Campanacci grade III, extraosseous extension, and ≥5 denosumab injections were associated with a size reduction of ≥5% (*p* < 0.1). Since high collinearity was observed between Campanacci grade III and extraosseous extension, we excluded extraosseous extension from the multivariate analyses. Multivariate analysis showed Campanacci grade III significantly predicted a size reduction of ≥5% (OR, 11.171; CI, 1.023–122.014). However, no significant factors were identified for a lesion shrinkage of ≥5 mm. For spinopelvic lesions, multivariate analysis was not performed because Campanacci grade III was the only candidate predictor. A longest tumor diameter of ≥7 cm was significantly related to lesion shrinkage of ≥5 mm (OR, 20; CI, 1.676–238.63), but no factors were found to be significantly related to a size reduction of ≥5%.

### 3.3. Subgroup Analyses of Predictive Factors by Recurrence Status

The results of additional analyses conducted to identify factors associated with reduction in the sizes of primary and recurrent lesions are presented in Table 5 and Table 6, respectively. Univariate analysis of primary lesions revealed that a spinopelvic location and Campanacci grade III were associated with a lesion size reduction of ≥5% (*p* < 0.1). Multivariate analysis identified Campanacci grade III as a significant predictor (OR, 5.781; CI, 1.181–28.297). Univariate analysis indicated that ABC-like change, a standard protocol, and the longest tumor diameters being ≥5 cm and ≥7 cm were associated with a primary lesion size reduction of ≥5 mm (*p* < 0.1). Multivariate analysis confirmed that an ABC-like change significantly predicted a primary lesion size reduction of ≥5 mm (OR, 11.936; CI, 1.074–132.69). In contrast, no significant associations were observed in the subgroup with recurrent lesions.

Table 7 summarizes the predictive factors for lesion shrinkage of GCTB in the overall cohort and each subgroup.

## 4. Discussion

Denosumab has been reported to reduce the size of GCTB lesions. However, the clinical factors associated with lesion shrinkage remain unclear. This study aimed to identify the predictors of a denosumab-induced GCTB lesion size reduction in different clinical settings. Campanacci grade III significantly predicts proportional shrinkage in the entire cohort and in those with an extremity lesion or a primary lesion, while ABC-like change was associated with an absolute reduction in all patients and those with a primary lesion. In addition, the longest tumor diameter of ≥7 cm predicted absolute shrinkage in all patients and the subgroup with a spinopelvic lesion. These findings may help clinicians provide more individualized patient counseling by better informing patients about the likelihood of tumor shrinkage based on their tumor characteristics. Understanding these predictors can help set realistic expectations for treatment response to neoadjuvant denosumab.

Several studies have evaluated GCTB response to treatment using RECIST criteria, which focuses on the longest diameter [7,8]. In previous studies, the rates of patients with a tumor size reduction of ≥30% were 25.1% (47 of 187) and 43.8% (35 of 80), respectively, whereas we found that 45.2% of patients (19 of 45) achieved a size reduction of ≥5%. This apparent discrepancy in response rates may be attributed to the shorter duration of denosumab treatment in the current study. The median number of denosumab administrations in our study was five, which contrasts with median numbers of 16 and 8, respectively, in the aforementioned studies [7,8]. Recently, there has been a trend to administer denosumab for shorter durations in the preoperative setting for GCTB, and three or fewer administrations are now referred to as ‘ultra-short-term’ use [13]. A study [13] reported no significant differences between clinical, histological, or radiological responses of patients who received denosumab ≤3 times or >3 times. However, although the tumor-reducing effect of denosumab has been documented in some patients with GCTB, no study has identified clinical predictors.

Our finding suggests that an ABC-like change might predict a reduction in post-denosumab GCTB lesion size. Aneurysmal bone cyst-like change, characterized by blood-filled cystic spaces, can be secondarily induced in 10–14% of all GCTB cases [14]. For these highly vascular lesions [13], the delivery of denosumab, which circulates in the bloodstream, may be elevated, potentially augmenting its therapeutic efficacy [15]. However, empirical evidence regarding the pharmacological mechanism underlying enhanced delivery is lacking.

Campanacci grade is assessed using plain radiographs, and grade III is distinguished from grades I and II by cortical disruption and extension into soft tissue, which suggests a more aggressive nature driven by the interaction between receptor activator of nuclear factor-kappa B and RANKL [4]. Consequently, Campanacci grade III GCTB may respond more to RANKL inhibitor. In our study, all GCTB patients classified as Campanacci grade III exhibited extraosseous extension by MRI, which introduced collinearity in the multivariate analysis. Therefore, extraosseous extension, which was statistically less significant, was excluded from the final model. Although advanced imaging techniques are essential for evaluating extraosseous extension, Campanacci grade can be more frequently assessed using radiographs alone. Thus, Campanacci grade could be considered the more clinically pragmatic variable.

The longest tumor diameter of >7 cm was identified as a significant predictor of an absolute reduction in GCTB size following denosumab treatment. This may have been due to a larger tumor volume allowing greater exposure of RANKL on stromal cells in the periphery of a GCTB to circulating denosumab, especially outside bone [2,3,16]. While a potential collinearity between Campanacci grade III and a longest tumor diameter of >7 cm, based on a perspective of orthopedic oncology, may have required exclusion of a variable among two, no collinearity was found between them. In this study, five GCTB lesions, classified as Campanacci grade III, had a tumor diameter of <7 cm and exhibited no measurable reduction in size after denosumab administration. This observation highlights the need for a more comprehensive assessment integrating multiple predictive factors rather than relying on individual parameters in isolation. Given that aggressive GCTBs exhibit rapid growth and concurrently invade and destroy cortical bone, our findings suggest that denosumab might be more effective at reducing the sizes of highly proliferative and invasive tumors.

This study has several limitations. First, the sample size of 45 patients was relatively small. In addition, the small number of those with recurrent GCTB made it difficult to identify statistically significant factors in this subgroup. Concerns about the potential adverse effects of denosumab treatment for GCTB, such as an increased risk of local recurrence and malignant transformation, were widely discussed during the study period [17,18], and may have caused clinicians to hesitate to use denosumab. Moreover, since denosumab is used as an adjunctive treatment rather than a standalone therapy, its administration is subject to the clinician’s preference. Some variables approached statistical significance. These variables may emerge as significant predictors in a larger cohort. To consider these potentially important variables, we applied a relatively relaxed criterion of *p* < 0.1 in the univariate analysis when selecting candidate predictors. Second, the criteria used to define size reduction were less strict than commonly used standards, such as RECIST criteria [7]. This adjustment was made due to the modest size reduction observed in our study population, which likely resulted from a relatively low median number of denosumab injections. However, the therapeutic value of denosumab treatment that is of interest in our study should ultimately be better surgical outcome; thus, we believe using a less strict criterion was clinically meaningful. Moreover, unlike the treatment of malignant tumors, where the primary goal is to reduce the lesion size, the primary goal of treating non-malignant tumors like GCTB is symptom relief, which means that criteria could differ from standards for malignant tumors like those recommended by RECIST [7]. Third, while GCTBs predominantly arise in the appendicular bone, our analysis included a high proportion (44%) of cases located in the spinopelvic region, which did not accurately represent its typical clinical distribution and may have induced some bias related to lesion location when determining predictive factors in all patients. Thus, we performed subgroup analyses to differentiate the spinopelvic and extremity groups. Finally, several variables, including the denosumab administration period and symptom duration, were excluded from the analysis due to missing data. This suggests the possibility that some meaningful predictors may not have been considered and the need for follow-up studies to address additional variables and ensure a more comprehensive analysis.

## 5. Conclusions

This study showed that several clinical factors, including Campanacci grade, the longest tumor diameter, and ABC-like changes, predicted post-denosumab GCTB lesion size reduction, and that these reductions may vary with lesion location and recurrence status. Recognizing these factors might help clinicians tailor treatment strategies in different clinical settings. Further studies are warranted to elucidate the pharmacological mechanisms by which denosumab interacts with clinical factors to influence lesion shrinkage in GCTB, including prospective validation of the predictive value of such factors, as well as investigations of potential molecular correlates such as RANKL expression levels.

## Figures and Tables

**Table 1:** Participants’ characteristics and post-denosumab lesion shrinkages of giant cell tumor of bone.

Characteristics	Overall Cohort, *n* (%)	Proportional Lesion Shrinkage, *n* (%)		Absolute Lesion Shrinkage, *n* (%)	
	(*n* = 45)	≥5% (*n* = 19)	<5% (*n* = 26)	≥5 mm (*n* = 15)	<5 mm (*n* = 30)
Sex					
Male	16 (35.6)	4 (25)	12 (75)	4 (25)	12 (75)
Female	29 (64.4)	15 (51.7)	14 (48.3)	11 (37.9)	18 (62.1)
Age, years					
<40	27 (60)	12 (44.4)	15 (55.6)	9 (33.3)	18 (66.7)
≥40	18 (40)	7 (38.9)	11 (61.1)	6 (33.3)	12 (66.7)
Lesion location					
Extremities	25 (55.6)	9 (36)	16 (64)	8 (32)	17 (68)
Spine/pelvis	20 (44.4)	10 (50)	10 (50)	7 (35)	13 (65)
Campanacci grade					
I	3 (6.7)	1 (33.3)	2 (66.7)	0 (0)	3 (100)
II	17 (37.8)	3 (17.6)	14 (82.4)	3 (17.6)	14 (82.4)
III	25 (55.6)	15 (60)	10 (40)	12 (48)	13 (52)
Aneurysmal bone cyst-like change					
Yes	15 (33.3)	8 (53.3)	7 (46.7)	8 (53.3)	7 (46.7)
No	30 (66.7)	11 (36.7)	19 (63.3)	7 (23.3)	23 (76.7)
Recurrence status					
Primary	35 (77.8)	14 (40)	21 (60)	10 (28.6)	25 (71.4)
Recurrent	10 (22.2)	5 (50)	5 (50)	5 (50)	5 (50)
Extraosseous extension					
Yes	27 (60)	14 (51.9)	13 (48.1)	11 (40.7)	16 (59.3)
No	18 (40)	5 (27.8)	13 (72.2)	4 (22.2)	14 (77.8)
Denosumab protocol compliance					
standard	30 (66.7)	13 (43.3)	17 (56.7)	12 (40)	18 (60)
non-standard	15 (33.3)	6 (40)	9 (60)	3 (20)	12 (80)
Total injection number, times					
<5	17 (37.8)	4 (23.5)	13 (76.5)	3 (17.6)	14 (82.4)
≥5	28 (62.2)	15 (53.6)	13 (46.4)	12 (42.9)	16 (57.1)
The longest tumor diameter, cm					
<5	23 (51.1)	8 (35.8)	15 (65.2)	5 (21.7)	18 (78.3)
≥5 and <7	9 (20)	3 (33.3)	6 (66.7)	2 (22.2)	7 (77.8)
≥7	13 (28.9)	8 (61.5)	5 (38.5)	8 (61.5)	5 (38.5)

Lesion shrinkage was defined as a decrease in the longest diameter measured in the axial view of MRI. The standard denosumab protocol was 120 mg every 4 weeks with additional doses of 120 mg on days 8 and 15 during the first month. A non-standard protocol was defined as any deviation from the standard regimen.

**Table 2:** Predictive factors for giant cell tumor of bone lesion size reduction.

Factors	Lesion Shrinkage of ≥5%				Lesion Shrinkage of ≥5 mm			
	Univariate Analysis		Multivariate Analysis		Univariate Analysis		Multivariate Analysis	
	OR (95% CI)	*p* Value	OR (95% CI)	*p* Value	OR (95% CI)	*p* Value	OR (95% CI)	*p* Value
Sex (Female vs. Male)	3.214 (0.837–12.346)	0.089	3.023 (0.699–13.073)	0.139	1.833 (0.472–7.126)	0.382		
Age (<40 years vs. ≥40 years)	1.258 (0.373–4.237)	0.712			1.0 (0.282–3.544)	1.000		
Lesion location (Spine/pelvis vs. Extremities)	1.776 (0.536–5.882)	0.347			1.144 (0.329–3.968)	0.832		
Campanacci Grade (III vs. I + II)	6.0 (1.545–23.3)	0.010	4.819 (1.121–20.714)	0.035 *	5.231 (1.219–22.45)	0.026	2.58 (0.426–15.626)	0.303
Aneurysmal bone cyst-like change (Yes vs. No)	1.974 (0.562–6.939)	0.289			3.755 (1.002–14.07)	0.050	8.734 (1.159–65.845)	0.035 *
Recurrence status (Recurrent vs. Primary)	1.5 (0.365–6.157)	0.574			2.5 (0.592–10.555)	0.212		
Extraosseous extension (Yes vs. No)	2.8 (0.78–10.052)	0.114			2.406 (0.623–9.287)	0.203		
Denosumab protocol compliance (standard vs. non-standard)	1.147 (0.325–4.045)	0.831			2.667 (0.619–11.493)	0.188		
Number of denosumab injections (≥5 times vs. <5 times)	4.242 (0.935–19.256)	0.054	2.27 (0.511–10.08)	0.281	3.5 (0.817–14.986)	0.091	3.249 (0.514–20.555)	0.211
The longest tumor diameter (≥5 cm vs. <5 cm)	3.055 (0.804–11.6)	0.304			3.0 (0.819–10.991)	0.097	0.889 (0.106–7.477)	0.914
The longest tumor diameter (≥7 cm vs. <7 cm)	3.055 (0.804–11.6)	0.101			5.714 (1.414–23.097)	0.014	12.380 (1.038–147.694)	0.047 *

Lesion shrinkage was defined as a decrease in the longest diameter measured in the axial view of MRI. A *p*-value of < 0.1 by univariate analysis was used to select candidate predictors. The standard denosumab protocol was 120 mg every 4 weeks with additional doses of 120 mg on days 8 and 15 during the first month. A non-standard protocol was defined as any deviation from the standard regimen. Statistically significant *p*-values were marked as *. OR = odds ratio; CI = confidence interval.

**Table 3:** Predictive factors of giant cell tumor of bone lesion size reduction for extremity lesions.

Factors	Lesion Shrinkage of ≥5%				Lesion Shrinkage of ≥5 mm			
	Univariate Analysis		Multivariate Analysis		Univariate Analysis		Multivariate Analysis	
	OR (95% CI)	*p* Value	OR (95% CI)	*p* Value	OR (95% CI)	*p* Value	OR (95% CI)	*p* Value
Sex (Female vs. Male)	2 (0.366–10.919)	0.423			1.481 (0.265–8.267)	0.654		
Age (<40 years vs. ≥40 years)	2 (0.366–10.919)	0.423			1.481 (0.265–8.267)	0.654		
Campanacci Grade (III vs. I + II)	13.333 (1.321–134.615)	0.028	11.171 (1.023–122.014)	0.048 *	10 (0.995–100.462)	0.050	4.912 (0.404–59.7)	0.212
Aneurysmal bone cyst-like change (Yes vs. No)	3.333 (0.599–18.543)	0.169			5.5 (0.836–36.198)	0.076	3.291 (0.402–26.926)	0.267
Recurrence status (Recurrent vs. Primary)	7.5 (0.645–87.193)	0.107			9.6 (0.807–114.173)	0.073	4.168 (0.286–60.727)	0.296
Extraosseous extension (Yes vs. No)	10.286 (1.030–102.753)	0.047			7.875 (0.788–78.671)	0.079		
Denosumab protocol compliance (standard vs. non-standard)	1.591 (0.239–10.572)	0.631			1.25 (0.185–8.444)	0.819		
Number of denosumab injections (≥5 times vs. <5 times)	5.833 (0.900–37.818)	0.064	4.573 (0.577–36.230)	0.150	4.286 (0.661–27.785)	0.127		
The longest tumor diameter (≥5 cm vs. <5 cm)	1.333 (0.254–7.007)	0.734			1.833 (0.333–10.095)	0.486		
The longest tumor diameter (≥7 cm vs. <7 cm)	2 (0.231–17.338)	0.529			2.5 (0.284–22.042)	0.409		

Lesion shrinkage was defined as a decrease in the longest diameter measured in the axial MRI view. A *p*-value of < 0.1 by univariate analysis was used to select candidate predictors. Extraosseous extension was excluded from the multivariate analysis due to collinearity with Campanacci grade III. The standard denosumab protocol was 120 mg every 4 weeks with additional doses of 120 mg on days 8 and 15 during the first month. A non-standard protocol was defined as any deviation from the standard regimen. Statistically significant *p*-values were marked as *. OR = odds ratio; CI = confidence interval.

**Table 4:** Predictive factors of giant cell tumor of bone lesion size reduction for spinopelvic lesions.

Factors	Lesion Shrinkage of ≥5%		Lesion Shrinkage of ≥5 mm	
	Univariate Analysis		Univariate Analysis	
	OR (95% CI)	*p* Value	OR (95% CI)	*p* Value
Sex (Female vs. Male)	6 (0.532–67.649)	0.147	2.667 (0.237–30.066)	0.427
Age (<40 years vs. ≥40 years)	1.556 (0.244–9.913)	0.640	1.687 (0.251–11.336)	0.590
Campanacci Grade (III vs. I + II)	3.5 (0.549–22.304)	0.185	2.917 (0.407–20.899)	0.407
Aneurysmal bone cyst-like change (Yes vs. No)	2.25 (0.170–29.767)	0.538	4.8 (0.35–65.758)	0.24
Recurrence status (Primary vs. Recurrent)	2.667 (0.361–19.608)	0.337	1.11 (0.148–8.333)	0.919
Extraosseous extension (Yes vs. No)	1 (0.167–5.985)	1.000	0.833 (0.129–5.396)	0.848
Denosumab Protocol Compliance (standard vs. non-standard)	1 (0.167–5.985)	1.000	7 (0.647–75.735)	0.109
Number of denosumab injections (≥5 times vs. <5 times)	1.714 (0.219–13.406)	0.608	2.667 (0.237–30.066)	0.427
The longest tumor diameter (≥5 cm vs. <5 cm)	2.333 (0.373–14.613)	0.365	7 (0.647–75.735)	0.109
The longest tumor diameter (≥7 cm vs. <7 cm)	3.5 (0.549–22.304)	0.185	20 (1.676–238.63)	0.018 *

Lesion shrinkage was defined as a decrease in the longest diameter measured in the axial MRI view. A *p*-value of <0.1 by univariate analysis was used to select candidate predictors. Multivariate analysis was not performed because Campanacci grade III was the only candidate predictor. The standard denosumab protocol was 120 mg every 4 weeks, with additional doses of 120 mg on days 8 and 15 during the first month. A non-standard protocol was defined as any deviation from the standard regimen. Statistically significant *p*-values were marked as *. OR = odds ratio; CI = confidence interval.

**Table 5:** Predictive factors of giant cell tumor of bone lesion size reduction for primary lesions.

Factors	Lesion Shrinkage of ≥5%				Lesion Shrinkage of ≥5 mm			
	Univariate Analysis		Multivariate Analysis		Univariate Analysis		Multivariate Analysis	
	OR (95% CI)	*p* Value	OR (95% CI)	*p* Value	OR (95% CI)	*p* Value	OR (95% CI)	*p* Value
Sex (Female vs. Male)	1.875 (0.441–7.963)	0.394			0.844 (0.185–3.804)	0.825		
Age (<40 years vs. ≥40 years)	1.538 (0.359–6.579)	0.562			1.4 (0.287–6.849)	0.677		
Lesion location (Spine/pelvis vs. Extremities)	3.333 (0.806–13.699)	0.097	4 (0.817–19.608)	0.087	1.776 (0.403–7.874)	0.447		
Campanacci Grade (III vs. I + II)	5 (1.147–21.796)	0.032	5.781 (1.181–28.297)	0.030 *	3.5 (0.727–16.848)	0.118		
Aneurysmal bone cyst-like change (Yes vs. No)	1.778 (0.403–7.844)	0.447			4 (0.824–19.423)	0.086	11.936 (1.074–132.69)	0.044 *
Extraosseous extension (Yes vs. No)	2.75 (0.651–11.624)	0.169			2.154 (0.451–10.287)	0.336		
Denosumab protocol compliance (standard vs. non-standard)	1.538 (0.359–6.599)	0.562			7.071 (0.774–64.575)	0.083	4.15 (0.353–48.761)	0.258
Number of denosumab injections (≥5 times vs. <5 times)	2.75 (0.651–11.624)	0.169			2.154 (0.451–10.287)	0.336		
The longest tumor diameter (≥5 cm vs. <5 cm)	2.167 (0.547–8.586)	0.271			4.148 (0.854–20.138)	0.078	1.231 (0.071–21.454)	0.887
The longest tumor diameter (≥7 cm vs. <7 cm)	3.187 (0.698–14.557)	0.135			7.875 (1.503–41.271)	0.015	12.461 (0.496–313.261)	0.125

Lesion shrinkage was defined as a decrease in the longest diameter measured in the axial MRI view. A *p*-value of < 0.1 by univariate analysis was used to select candidate predictors. The standard denosumab protocol was 120 mg every 4 weeks, with additional doses of 120 mg on days 8 and 15 during the first month. A non-standard protocol was defined as any deviation from the standard regimen. Statistically significant *p*-values were marked as *. OR = odds ratio; CI = confidence interval.

**Table 6:** Predictive factors of giant cell tumor of bone lesion size reduction for recurrent lesions.

Factors	Lesion Shrinkage of ≥5%		Lesion Shrinkage of ≥5 mm	
	Univariate Analysis		Univariate Analysis	
	OR (95% CI)	*p* Value	OR (95% CI)	*p* Value
Sex (Female vs. Male)	NE	0.999	NE	0.999
Age (<40 years vs. ≥40 years)	1 (0.080–12.557)	1.000	1 (0.08–12.557)	1.000
Lesion location (Extremities vs. Spine/Pelvis)	6 (0.354–101.568)	0.214	6 (0.354–101.568)	0.214
Campanacci Grade (III vs. I + II)	NE	0.999	NE	0.999
Aneurysmal bone cyst-like change (Yes vs. No)	2.25 (0.179–28.254)	0.530	2.25 (0.179–28.254)	0.530
Extraosseous extension (Yes vs. No)	2.667 (0.158–45.141)	0.497	2.667 (0.158–45.141)	0.497
Denosumab protocol compliance (non-standard vs. standard)	0.375 (0.022–6.348)	0.497	0.375 (0.022–6.348)	0.497
Number of denosumab injections (≥5 times vs. <5 times)	NE	0.999	NE	0.999
The longest tumor diameter (≥5 cm vs. <5 cm)	1 (0.08–12.557)	1.000	1 (0.08–12.557)	1.000
The longest tumor diameter (≥7 cm vs. <7 cm)	2.667 (0.158–45.141)	0.497	2.667 (0.158–45.141)	0.497

Lesion shrinkage was defined as a decrease in the longest diameter measured in the axial view of MRI. Univariate analysis selected candidate predictors of *p* < 0.1. Multivariate analysis was not performed since there were fewer than two candidate predictors. The standard denosumab protocol was 120 mg every 4 weeks with additional doses of 120 mg on days 8 and 15 during the first month. A non-standard protocol was defined as any deviation from the standard regimen. OR = odds ratio; CI = confidence interval; NE = not estimable due to model non-convergence.

**Table 7:** Summary of predictive factors for giant cell tumor of bone lesion size reduction in overall and subgroup analyses.

Factors	Lesion Shrinkage of ≥5%	Lesion Shrinkage of ≥5 mm
All patients	Campanacci grade III	ABC-like change The longest diameter ≥7 cm
Subgroup by lesion location		
Extremities	Campanacci grade III	
Spine/pelvis		The longest diameter ≥7 cm
Subgroup by recurrence status		
Primary	Campanacci grade III	ABC-like change
Recurrent		

Lesion shrinkage was defined as a decrease in the longest diameter measured in the axial view of MRI.

## Data Availability

The data may be available from the corresponding author based on reasonable request.
